# Awareness About Keratoconus and Its Relation With Eye Rubbing: A Cross-Sectional Study in Medina

**DOI:** 10.7759/cureus.32030

**Published:** 2022-11-29

**Authors:** Essam S Kordi, Amirah M Almokhtar, Esraa K Alshareef, Aaesha A Alkayyal, Jana O Alharbi, Abdulrahman H Alharbi

**Affiliations:** 1 Ophthalmology, Ohud Hospital, Medina, SAU; 2 Medicine, Taibah University, Medina, SAU; 3 Medicine, Vision Colleges, Riyadh, SAU

**Keywords:** cornea, awarness, allergy, eye rubbing, keratoconus

## Abstract

Background: Keratoconus is a non-inflammatory, bilateral, asymmetrical progressive disorder characterized by ectasia, thinning, and increased curvature of the cornea, as well as loss of visual acuity. Eye rubbing is considered the most common risk factor for keratoconus.

Objectives: This study aims to assess the awareness of the population in Medina about keratoconus and its relation to eye rubbing.

Methods: This is a cross-sectional study involving 767 participants via an online pre-designed questionnaire from November 2021 to January 2022, in Medina, Saudi Arabia.

Results: Among the study participants, 94.1% have a lack of awareness about keratoconus and its relation to eye rubbing. Participants who have a visual disturbance and positive family history of keratoconus were found to have good awareness levels. Those who heard about keratoconus represent 39.8% of the participants, and relatives with keratoconus were the most common source of their information. An allergic reaction was reported by 34.9% of the participants, and 7.7% have a family history of keratoconus. Only 27.8% believe in the relationship between keratoconus and allergy, and 61.9% have no idea about the treatment. For eye rubbing, 28.9% of participants believe it can lead to keratoconus; also, 80.4% reported rubbing their eyes, and itching was the most common cause of their behavior.

Conclusion: The majority of the participants have a lack of awareness about keratoconus and its relation to eye rubbing. Health education programs for the population should be conducted to enhance public awareness about keratoconus.

## Introduction

Keratoconus (KC) was first described in 1854 by Nottingham and is a non-inflammatory, bilateral, asymmetrical, progressive disorder characterized by ectasia, thinning, increased curvature of the cornea, and loss of visual acuity, especially with high irregular astigmatism [1,2].

The prevalence of KC is estimated to be 1.38 per 1000 people [3]. Asian or Middle Eastern patients with KC are also younger at diagnosis and have more severe disease presentations, according to previous studies [4]. The increased prevalence of KC in warmer, sunnier countries compared with those in Europe and North America has led to the theory that intense sunlight in these areas is a causative factor in genetically susceptible individuals [5].

However, the exact underlying cause of this illness is still unknown. Many possible mechanisms, including those of biochemical, genetic, and mechanical origin, have been examined, and a multifactorial origin is frequently mentioned [6]. Environmental variables, including eye rubbing, atopy, and UV exposure, appear to be triggers for KC in genetically susceptible individuals [7]. Ethnicity has a role as well, with Asians developing KC earlier and in a more aggressive form than Caucasians [8]. Furthermore, despite being visually asymptomatic, patients with a positive family history of KC have been demonstrated to exhibit early topographical changes suggestive of KC [8]. Increased disease concordance has also been found in monozygotic twin and familial investigations [8].

Eye rubbing, a frequent behavior that begins before sleep and lasts the entire day as a reaction to eye irritation, exhaustion, and emotional stress [9], is implicated as a significant exogenous environmental factor that induces a mechanical change in the cornea, often as the second hit in a double-hit hypothesis [10]. Abnormal eye rubbing can occur subsequent to annoying symptoms, such as dryness and irritation [8]. Atopy and allergies are the most prevalent risk factors for the persistent behavior of inappropriate eye rubbing [7]. According to several studies, KC and atopy are linked because pruritus increases eye rubbing, which causes corneal mechanical wear and progressive ectasia [11-13]. 

In a retrospective Saudi study conducted among KC patients in King Khaled Hospital, it was found that 44.8% of patients rubbed their eyes. Additionally, it was noted that eye rubbing was the most common risk factor, accounting for 100% of all cases [14]. In addition, according to a recent study, eyes with KC respond differently to eye rubbing than normal eyes, exhibiting significant increases in posterior astigmatism, intraocular pressure, and anterior chamber volume following eye rubbing [15]. 

A study was conducted by Al-Amri et al. on 374 female and 19 male nonmedical students in Abha city to measure the level of KC awareness [16]. The majority of those who were not aware of KC were female (95.7%) and between the ages of 17 and 21 (68.3%). Moreover, 355 (90.3%) denied the relationship between KC and allergic eye disease [16]. According to a recent survey conducted by Alnahdi et al. in Jeddah, 48.8% of the Saudi population had heard about KC, with reading and lectures ranking as the most common sources of information (18.3%) [17]. According to 32.9% and 50.4% of the respondents, KC is associated with allergies and myopia, respectively. In the study, 75.8% of people rubbed their eyes, and eye itching was the most common reason for the majority of them (40.9%). According to one-third of the participants, eye rubbing can cause KC (34.3%) [17].

Unless the posterior and anterior corneal surfaces are assessed using corneal tomography in the early stages of the disease, the problem may go unnoticed [9]. KC is the most common indication for penetrating keratoplasty in the developed world, with advanced patients requiring corneal transplantation [6]. This study aims to assess the awareness of KC in the population of Medina and its relation to eye rubbing.

## Materials and methods

 Study design and setting

A cross-sectional quantitative study targeting the residents of Medina, Saudi Arabia was conducted to determine the awareness of KC. Ethical approval was obtained from the Institutional Review Board at the General Directorate of Health Affairs in Medina (approval number: H-03-M-084). The study followed the directives of the Helsinki Declaration in all stages.

The questionnaire was distributed via using personal contacts and social media platforms such as Twitter (Twitter, Inc., San Francisco, California, United States), WhatsApp (WhatsApp LLC, Menlo Park, California, United States), and Instagram (Meta Platforms, Inc., Menlo Park, California, United States) from November 2021 to January 2022, targeting the residents of Medina province. The aim of the study was explained clearly to the participants, and informed consent was obtained before starting the online questionnaire. Voluntary participation was ensured in this study, and all personal identities were kept confidential. The included participants were residents of Medina province who were over 18 years old. Respondents who provided incomplete or suspected incorrect data such as repeating the same answer to all questions were excluded from the study.

 Survey instrument

We used a pre-designed questionnaire after obtaining consent from the corresponding author of a previous study conducted in Jeddah, Saudi Arabia [17]. The questionnaire was pretested in a pilot study on 15 participants to ensure the clarity of the questionnaire and identify any omissions; several additions and modifications were made. We divided the questionnaire into three parts: the first included questions regarding patients’ demographic data, such as age, gender, and level of education. The second included three Yes/No questions to determine the presence of eye allergies and diseases. In particular, it asked whether participants had suffered from allergies. This part included (i) eye allergies, (ii) skin allergies, (iii) gastrointestinal allergies, and (iv) chest allergies. Participants were then asked whether they had suffered from eye diseases or eyesight distress. This part included the following types: (i) farsightedness/nearsightedness, (ii) KC, (iii) use of contact lenses, (iv) history of refractive surgery, and (v) history of other eye surgeries. The last question was ‘Do you have a family history of KC?‘. All participants answered each of the three questions. The third part included nine questions to assess the awareness of KC, its etiology and treatment, as well as the reasons for eye rubbing. The participants were asked to choose the most appropriate reason from a list based on their opinions and knowledge.

Data analysis

After extraction, the data were revised, coded, and entered into the statistical software IBM SPSS Statistics for Windows, Version 22.0 (Released 2013; IBM Corp., Armonk, New York, United States). Two-tailed tests were used for all statistical analyses. A p<0.05 indicated statistical significance. For knowledge questions, each correct response was worth one point, and the sum of the discrete scores for all questions was calculated. Poor awareness was defined as a score of less than 60% of the total score, and good awareness was defined as a score of at least 60%. All variables, including the participants’ biographical data, their family history of KC, their medical history of eye illnesses, and their source of knowledge regarding KC, underwent descriptive analysis based on frequency and percent distribution. Also, participants’ awareness regarding KC and its risk factors and treatment methods, as well as the overall awareness level excluding the practice of eye rubbing, was assessed in frequency tables and graphs. Cross-tabulation was used to assess the distribution of participants’ awareness levels according to their personal data and practice. Relations were tested using the Pearson chi-square test and an exact probability test for small frequency distributions.

## Results

A total of 767 participants completed the study questionnaire. As shown in Table 1, the participants’ ages ranged from 18 years to 65 years with a mean age of 26.4 ± 12.8 years. A total of 613 (79.9%) participants were females. Regarding educational level, 173 (22.6%) had a secondary degree, whereas 560 (73%) had a university degree, and 34 (4.4%) were below the secondary level.

**Table 1:** Sociodemographic data of study participants

Personal data	Number	Percentage
Age in years		
18-30	461	60.1%
31-40	194	25.3%
41-50	83	10.8%
> 50	29	3.8%
Gender		
Male	154	20.1%
Female	613	79.9%
Educational level		
Below secondary	34	4.4%
Secondary	173	22.6%
University and higher	560	73.0%

Table 2 shows the medical and family history of eye diseases among study participants. A total of 268 (34.9%) participants reported complaining of an allergic disorder, which was eye allergy among 39.1% of them, followed by skin allergy (36.4%), chest allergy (29.6%), and gastrointestinal tract allergy (12.2%). A history of visual/eye disorders was reported among 414 (54%) participants. The most reported were refractive error (78.7%), followed by the use of medical lenses (20.3%), KC (13.2%), and history of refractive error (RE) surgery (11%). A family history of KC was reported among 59 (7.7%) participants.

**Table 2:** Medical and family history of eye diseases among study participants RE: refractive error

Family and medical history	Number	Percentage
Had allergy		
Yes	268	34.9%
No	499	65.1%
Type of allergy		
Chest allergy	87	29.6%
Skin allergy	107	36.4%
Eye allergy	115	39.1%
Nasal allergy	18	6.1%
Gastrointestinal tract allergy	36	12.2%
Others	17	5.8%
Had visual/eye problems?		
Yes	414	54.0%
No	353	46.0%
What is the disorder?		
Refractive error	322	78.7%
Keratoconus	54	13.2%
Use visual lenses	83	20.3%
Surgery for RE	45	11.0%
Eye surgery	5	1.2%
Amblyopia	5	1.2%
Others	5	1.2%
Family history of keratoconus		
Yes	59	7.7%
No	329	42.9%
Don't know	379	49.4%

Table 3 shows public awareness and perception of KC in Medina, Saudi Arabia. Of the study participants, 39.8% had heard about KC. The majority of them reported relatives with KC as their primary information source (27.5%), followed by lectures and reading (26.9%), social media (24.3%), and physicians (19%). A total of 14.9% know that KC is a decrease in corneal thickness, 27.8% think that there is a relationship between KC and allergy, and 42.8% reported that KC led to visual impairment. As for treatment methods of KC, 26.7% reported surgery, 11.7% know about wearing medical glasses, and 8.9% mentioned medical lenses. A total of 28.9% of the study participants agreed that frequent eye rubbing is a habit that may lead to KC, 3.1% think it is a harmful habit in general, while 6.8% think it is a safe habit.

**Table 3:** Awareness and perception of keratoconus among study participants

Awareness items	Number	Percentage
Heard about keratoconus		
Yes	305	39.8%
No	462	60.2%
Source of information regarding keratoconus		
Relative with keratoconus	84	27.5%
Lectures/reading	82	26.9%
Social media	74	24.3%
Physician	58	19.0%
I am a case	6	2.0%
Friends	1	0.3%
What is keratoconus?		
Thinning of corneal thickness	114	14.9%
Increased corneal thickness	85	11.1%
Corneal inflammation	58	7.6%
Don't know	510	66.5%
Is there a relationship between keratoconus and allergy?		
Yes	213	27.8%
No	83	10.8%
Don't know	471	61.4%
Does keratoconus lead to visual impairment?		
Yes	328	42.8%
No	7	0.9%
Don't know	432	56.3%
Treatment method of keratoconus		
Surgery	205	26.7%
Medical glass	90	11.7%
Medical lenses	68	8.9%
Eye drops	52	6.8%
No treatment	29	3.8%
Don't know	475	61.9%
Frequent eye rubbing is		
A habit that may lead to keratoconus	222	28.9%
A habit that may harm the eye	24	3.1%
It may cause allergy/itch	3	0.4%
A safe habit	52	6.8%
Don't know	466	60.8%

**Figure 1:**
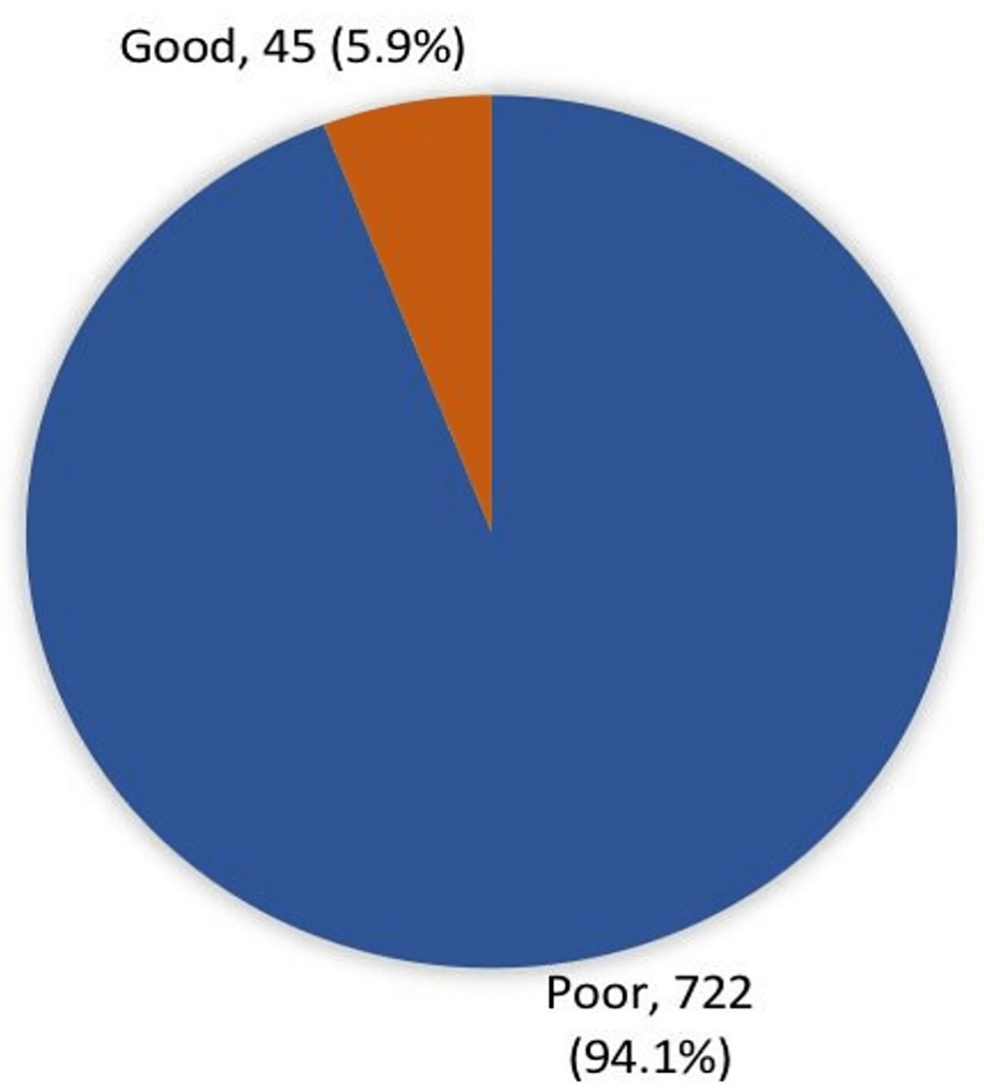
Overall awareness about keratoconus among study participants

Figure 1 shows overall awareness regarding KC among study participants. Seven hundred twenty-two (94.1%) study participants had poor awareness levels regarding KC, while only 45 (5.9%) had good awareness levels.

**Figure 2:**
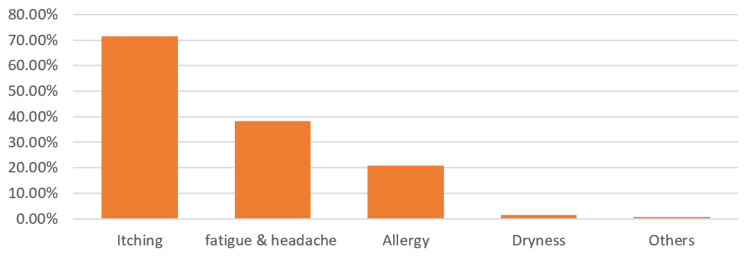
Causes for eye rubbing reported by study participants (%)

Figure 2 shows the causes of eye rubbing most reported by the study participants. A total of 617 (80.4%) participants reported rubbing their eyes. The causes reported the most were itching sensation (71.6%), fatigue and headache (38.2%), allergy (20.8%), and dryness sensation (1.6%). 

Table 4 shows the overall awareness regarding KC according to the sociodemographic data and medical history of the participants. Good awareness was detected among 7.5% of participants with visual problems compared to 4% of others but this was not of statistical significance (P=0.039). Also, 20.3% of participants with a family history of KC had good awareness regarding the disease versus 6.4% of those with negative family history (P=0.001). All other factors were insignificantly associated with participants' awareness levels.

**Table 4:** Overall awareness of the participants regarding keratoconus according to their sociodemographic data and medical history *A p-value less than 0.05 is considered statistically significant

Factors	Awareness level				p-value*
	Poor		Good		
	No	%	No	%	
Age in years					0.887
18-30	432	93.7%	29	6.3%	
31-40	184	94.8%	10	5.2%	
41-50	78	94.0%	5	6.0%	
> 50	28	96.6%	1	3.4%	
Gender					0.989
Male	145	94.2%	9	5.8%	
Female	577	94.1%	36	5.9%	
Educational level					0.346
Below secondary	33	97.1%	1	2.9%	
Secondary	166	96.0%	7	4.0%	
University and higher	523	93.4%	37	6.6%	
Had allergy					0.380
Yes	255	95.1%	13	4.9%	
No	467	93.6%	32	6.4%	
Had visual/eye problems?					0.039
Yes	383	92.5%	31	7.5%	
No	339	96.0%	14	4.0%	
Family history of keratoconus					0.001
Yes	47	79.7%	12	20.3%	
No	308	93.6%	21	6.4%	
Don't know	367	96.8%	12	3.2%	
Do you rub your eyes?					0.642
Yes	582	94.3%	35	5.7%	
No	140	93.3%	10	6.7%	

## Discussion

Recently, awareness of KC has increased, and patients have a better quality of life knowing that their disorder can be treated appropriately [18]. More people are being diagnosed with KC in the early stages of the disease with available approved treatment options, such as corneal cross-linking for progressive KC and lenses [19]. Today, patients can take advantage of appropriate treatment options faster, which helps to preserve vision and allows them to continue their normal life [20]. The patient is often asymptomatic in the early stages of the condition; visual acuity declines as the condition worsens, and finally, the patient experiences severe vision loss and visual distortion. Hence, awareness of KC is crucial.

The current study aimed to assess the awareness about KC and its relation to eye rubbing in the population in Medina. The study showed that public awareness regarding KC is very low, and the vast majority of people are unaware of the condition. However, those with high levels of education have an awareness level of 6%, which may be attributed to the fact that these individuals read more and enhance their general knowledge. More than one-third of the study participants reported having heard about KC. The most reported source of information was relatives with KC (25%), lectures/reading (25%), social media (25%), and physicians (19%). A total of 14.9% knew that KC is a thinning of the cornea, while 27.8% thought that there was a relationship between KC and allergy, and less than half of them (42.8%) agreed that KC leads to visual impairment. In all, 26.7% chose surgery as a treatment method for KC, whereas 11.7% chose to wear prescription glasses, and 8.9% chose medical lenses. Of the study participants, 28.9% agreed that frequent eye rubbing is a habit that may lead to KC, 3.1% considered it a harmful habit in general, and 6.8% considered it a safe habit. In a study by Al-Amri et al., it was found that 57.5% of non-medical students had never heard about KC; moreover, only 8.1% had learned about KC from their doctor and 60.6% did not know what KC was [16]. Only 24.2% answered that it was thinning of the cornea, while 33.6% knew that KC leads to myopia and astigmatism. More than 90% ignored the association between KC and allergic eye disease. About one-fifth thought that KC had a hereditary background. About 88% of the study participants did not know about KC treatment options [16].

Another study by Alruwaili et al. estimated the mean knowledge score for KC among the Saudi population at 4.12 ± 2.6; 67.5% of the participants had low scores [21]. Knowledge scores were not found to be statistically significantly correlated with any of the sociodemographic data. In all, 76% and 42.5% of those surveyed agreed that a hereditary predisposition and persistent ocular inflammation may predispose them to KC, respectively. In all, 38.1% of the participants reported that KC can be treated with spectacles or contact lenses in its early stages [21]. In Riyadh, Alkadi et al. found that the majority of the sample (38.4%) had a high level of knowledge about KC, 31.3% had a moderate level of knowledge and 30.3% had a low level of knowledge [22]. Moreover, 30% of participants reported that they had acquired their knowledge of KC from the internet, while 29% stated that they had no knowledge of KC at all. Similar results were found in Saudi Arabia's urban community, where awareness of particular eye illnesses was noticeably low [23]. Young age and female gender were significantly associated with poor awareness in our study. Similar findings were reported among Hail University and Taif University students [24,25]. In our community, women are mostly housewives, which makes information from their surroundings less accessible to them, and this could explain our findings. Moreover, young people are more interested in social media than in taking additional courses and lectures about common diseases in the community, which may also explain our results. Findings from developed countries showed notable awareness and knowledge gaps among the general population [26-28].

Most of the study participants reported rubbing their eyes, mainly due to itching, fatigue, headache, and allergy. In a retrospective Saudi study, conducted among KC patients in King Khaled Hospital, they found that 44.8% of patients rubbed their eyes. Additionally, it was noted that eye rubbing was the most prevalent risk factor [14]. Furthermore, according to a recent study, eyes with KC respond differently to eye rubbing than normal eyes, exhibiting significant increases in posterior astigmatism, intraocular pressure, and anterior chamber volume following eye rubbing [15]. Najmi et al. conducted a systematic review and found that keratocytes became thinner as a result of eye rubbing, and the intensity and length of rubbing affect the level of impact [9]. To prevent KC, it is recommended to avoid rubbing the eyes. This can be achieved by treating dry eyes, avoiding contact lens use, and preventing itchiness.

Our study is the first in the Medina region to discuss awareness of KC and its relation to eye rubbing. The study had some limitations, such as the distribution of the survey online through social media applications. As a result, it may not be representative of the entire population. Furthermore, because the participants were asked to self-report, our study may have faced recall bias.

## Conclusions

The vast majority of the study participants in Medina had a lack of awareness about KC and the effect of eye rubbing on it. As KC is common in the Kingdom of Saudi Arabia, we need to enhance public health awareness by conducting health education programs. Therefore, more research is required with a larger sample of a diverse population from Medina to obtain more reliable results.
